# Nontraumatic intrathoracic liver incarceration

**DOI:** 10.1016/j.radcr.2024.09.130

**Published:** 2024-10-23

**Authors:** Andrius Meskauskas, Claire Goumard

**Affiliations:** Department of Hepatobiliary Surgery and Liver Transplantation, Sorbonne Université, Hôpital Pitié-Salpêtrière, Assistance Publique - Hôpitaux de Paris, Paris, France

**Keywords:** Diaphragmatic hernia, Emergency surgery, Prenatal screening, Malformations, Pediatric surgery

## Abstract

Diaphragmatic hernias are a rare finding in adult population, especially in urgent settings. Nevertheless, the acute symptoms can be life-threatening and may require urgent surgical intervention. We report a rare case of a nontraumatic diaphragmatic hernia in a young adult with a history of laparotomy at the age of 1, suggesting a pre-existing malformation. The clinical presentation was dominated by respiratory symptoms and biological signs of hepatic ischemia, with no abdominal pain. Computed tomography demonstrated protrusion of the entire liver into the thoracic cavity. In this context, an emergency laparotomy was performed. The liver was reintroduced to the abdominal cavity, and the diaphragmatic defect repaired using a synthetic mesh, resulting in resolution of symptoms and a smooth postoperative recovery. Our case highlights the need for efficient diagnosis of the diaphragmatic hernias in adults, even without the context of trauma. A prompt and adequate repair of the defect, usually interposing a synthetic mesh, allows for complete and relatively fast recovery.

## Introduction

Diaphragmatic hernias are described as a protrusion of abdominal contents into the thoracic cavity due to a defect in the diaphragm. This condition results in the compression of the lungs, mediastinum and potential strangulation of herniated organs. These hernias are generally congenital, affecting up to 1 in 2000 pregnancies. They can be classified as posterolateral, anterior, or central, with the posterolateral variant (Bochdalek hernia) accounting for 75% of cases [1].

In the adult population, diaphragmatic hernia is a rare finding and is usually traumatic, concerning up to 2% of the patients admitted for blunt thoraco-abdominal trauma [2]. Accidental findings in adult are even rarer, representing less than 0.2% of CT-scans performed [3]. Interestingly, congenital hernias are typically left-sided [4] due to the faster closure of the right pleuroperitoneal canal. However, accidental findings are usually right-sided, likely because of the interposition of the liver protecting the intra-abdominal organs from ascending into the thoracic cavity [3].

The clinical presentation of diaphragmatic hernias varies significantly with the age of diagnosis. In infants, symptoms are mostly acute and result from pulmonary hypoplasia due to pulmonary compression [5]. Despite significant progress in the past 2 decades, the mortality rate of newborns with congenital diaphragmatic hernia remains around 30% [6]. Survivors face a high risk of pulmonary hypertension, lung hypoplasia, gastrointestinal and even neurological sequalae [7].

In contrast, congenital diaphragmatic hernias in adults are usually asymptomatic, and acute presentations are rare, leading to significant number of incidental discoveries. Despite the differences in presentation and consequences of diaphragmatic hernia between adults and newborns, surgical treatment remains gold standard in both cases in order to avoid further complications [5,8].

We repot a rare case of a nontraumatic right-sided diaphragmatic hernia in an adult that required urgent surgical intervention.

## Observation

A 24-year-old male presented to the emergency department with dyspnea and acute right hemithorax pain. He reported no history of trauma or specific medical history, except for a laparotomy at the age of 1 due to reported intestinal occlusion, without any documentation available initially.

Upon examination, the patient exhibited dyspnoea and tachycardia at 145 bpm without hypotension or abdominal pain. Auscultation found the absence of breath sounds on the right-side.

**Fig. 1:**
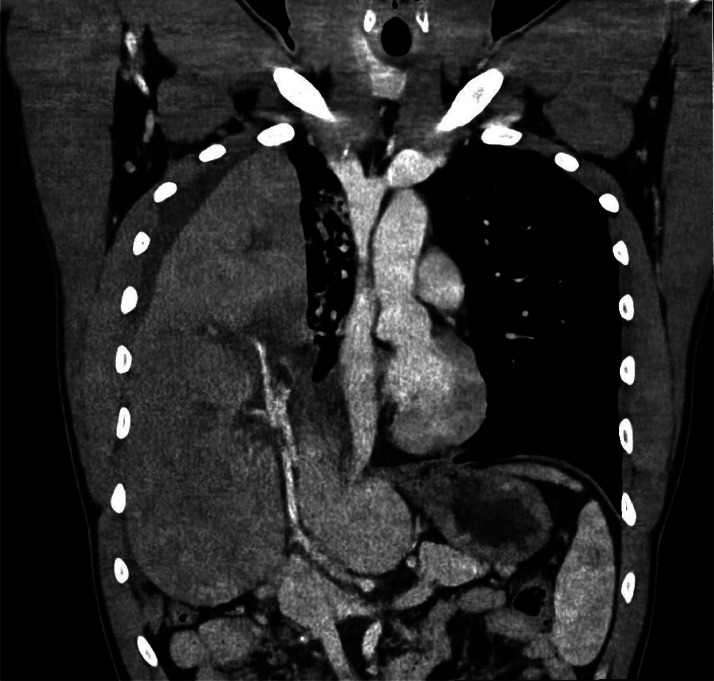
Coronal image from a portal venous CT, showing a complete ascension of the liver into the thoracic cavity, with the left lobe being completely verticalized; signs of hepatic hypoperfusion can also be identified.

Laboratory investigations found a total bilirubin level of 30 µmol/L, hepatic cytolysis at 10N, and a prothrombin level of 47%, suggesting hepatic ischemia. The CT-scan revealed a total ascent of the liver into the thorax resulting in right lung atelectasis and mediastinal deviation (Fig. 1).

**Fig. 2:**
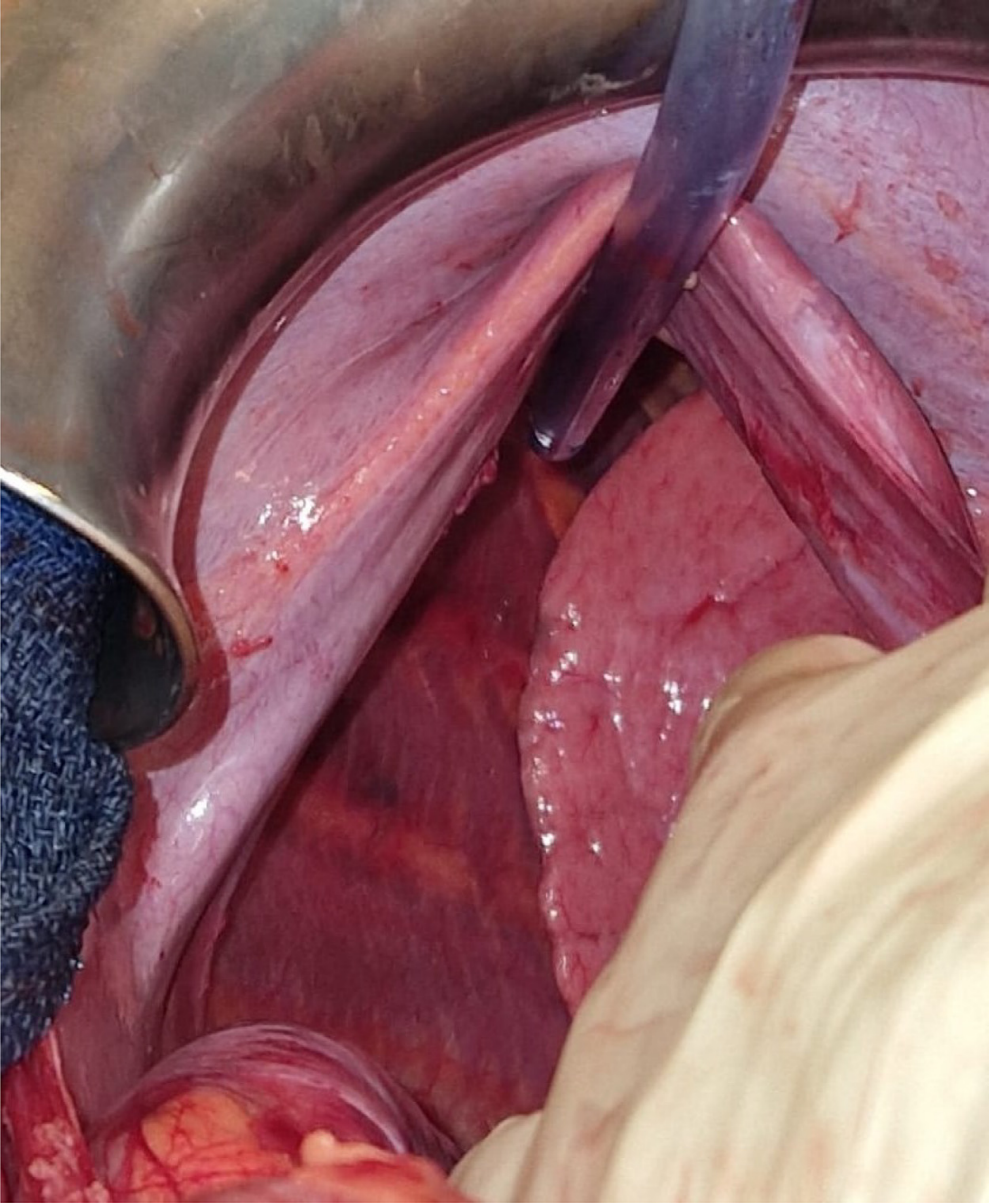
Preoperative view of the diaphragmatic hernia, the suction tube reclining the diaphragm and showing the right lung behind it.

**Fig. 3:**
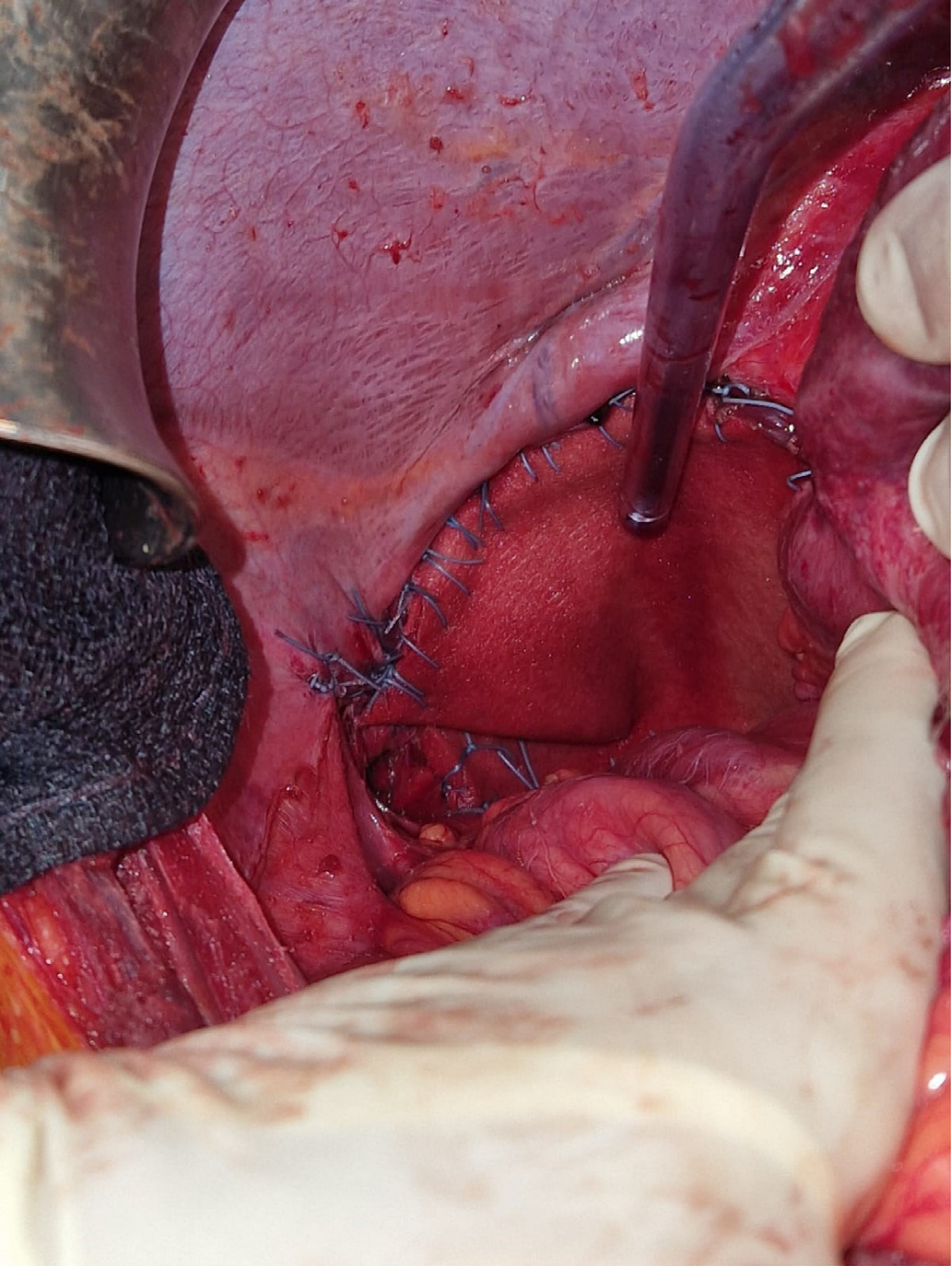
Preoperative view showing a nonresorbable mesh, fixed to the diaphragm anteriorly and to the ribs posteriorly with nonresorbable suture.

Due to worsening respiratory failure and hepatic ischemia, emergency laparotomy was performed. Intraoperatively, a right posterior hemi diaphragmatic defect was identified, supporting the diagnosis of a Bochdalek hernia (Fig. 2). The ischemic liver was reintroduced to the abdominal cavity and a nonresorbable mesh was used to reconstruct the diaphragm. The mesh was sutured to the atrophic diaphragm anteriorly and to the ribs posteriorly (Fig. 3).

The postoperative course was uneventful, involving noninvasive ventilation. Hepatic and respiratory functions normalized rapidly, leading to the patient's discharge on postoperative day 11.

**Fig. 4:**
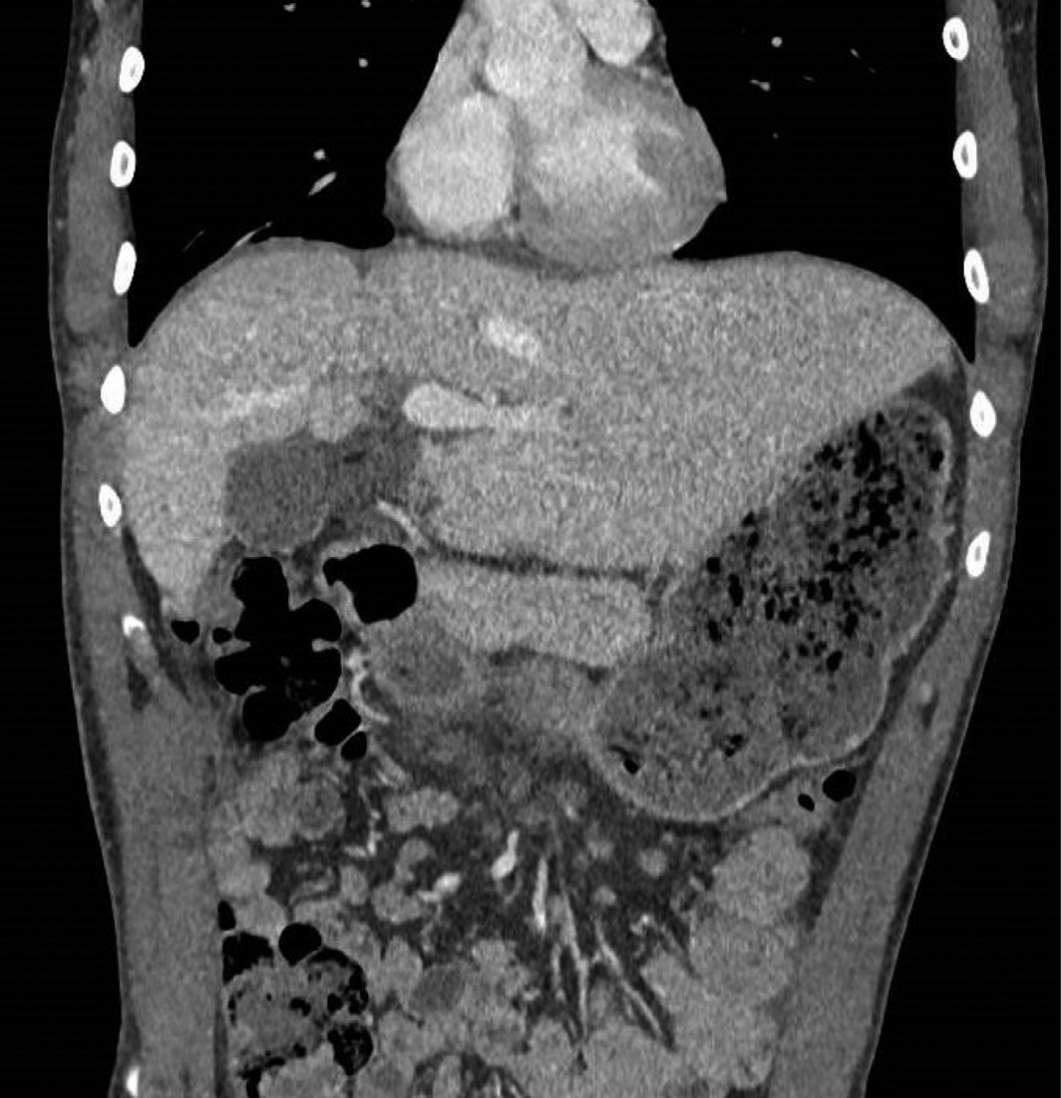
Thoracoabdominal CT scan at postoperative day 11 showing notable hypotrophy of the right liver.

**Fig. 5:**
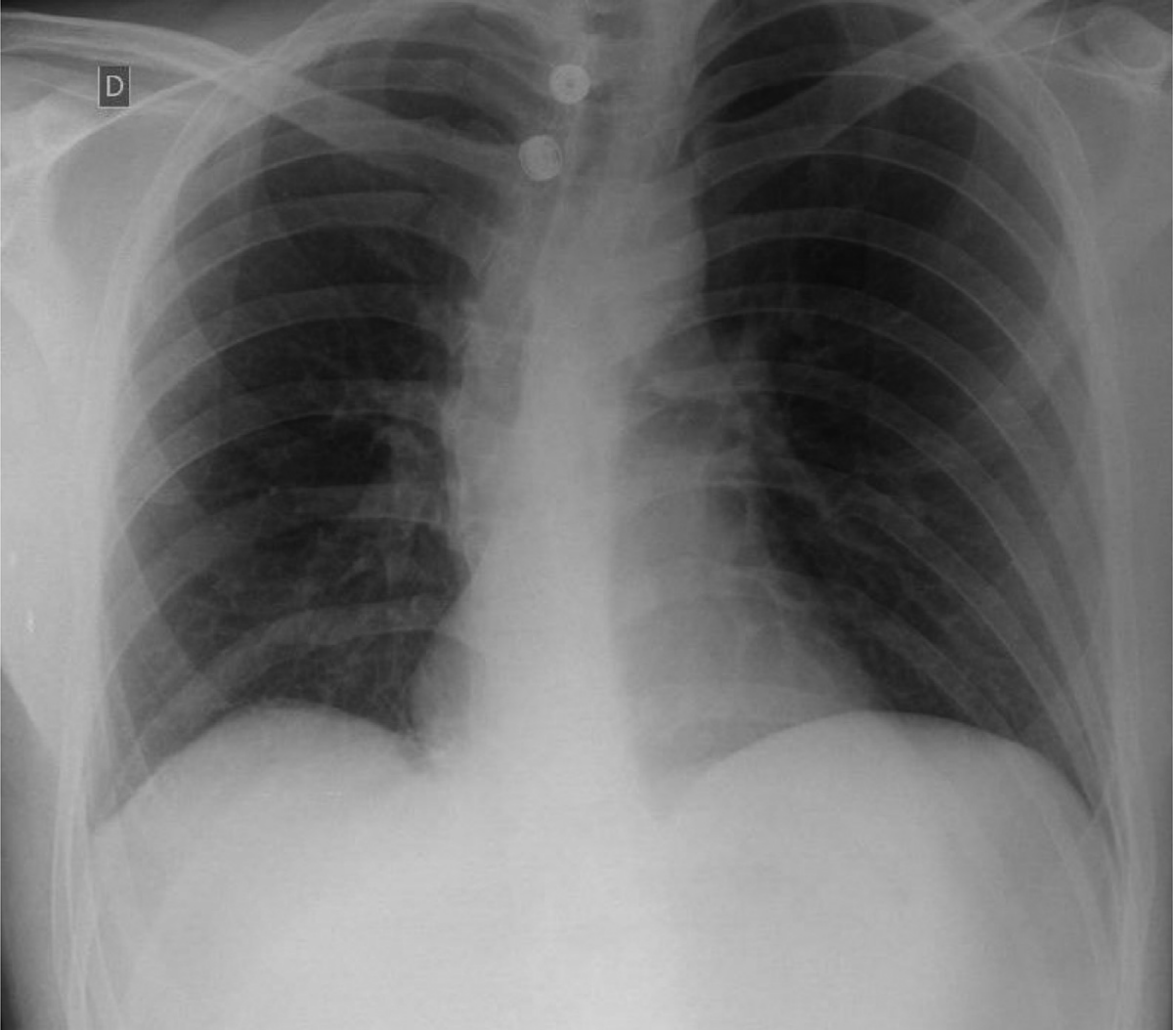
Postoperative X-ray 1 month after surgery.

Postoperative imaging was performed to ensure that there was no early recurrence of the hernia, and that the liver was being perfused correctly. A thoracoabdominal CT scan (Fig. 4) was performed on postoperative day 11, just before discharge from the hospital, and showed no complications or recurrence. Interestingly, hypotrophy of the right liver was observed, suggesting chronic subluxation of the liver prior to the onset of acute symptoms. A chest X-ray (Fig. 5), performed 1 month after the surgery was also considered satisfactory.

Paediatric medical records were retrieved later, indicating an intestinal occlusion caused by a spontaneous right colon volvulus at the age of 1. The absence of the right triangular ligament of the liver as well as of the right Toldt ligament were described, suggesting a pre-existent malformation. There were no precisions available concerning the right diaphragm. A single follow-up visit at the age of 5 reported a normal chest X-ray.

## Discussion

The case we present is a rare and complex occurrence, highlighting the need for efficient and sometimes urgent treatment. It is particularly unique due to its acute presentation in an adult, and the absence of abdominal pain.

A recent review by Ramspott et al. [8] in 2023 analyzed data on right-sided Bochdalek hernias in adults in 2023, identifying 44 patients. 61% of them were women. The most frequent symptom was dyspnoea (n = 21), followed by abdominal pain. The colon was the most commonly herniated organ (n = 23, 52%), with the liver involved in 12 cases (27%). In this review, 86% of the patients were treated surgically, predominantly via an abdominal approach. A surgical mesh was used in 43% of the cases. The rate of hernia or intervention-related complications was of 43% and 4 patients (9%) died.

Although it is generally accepted that diaphragmatic hernias in adults need surgical repair regardless of symptoms, the rarity of this condition limits the elaboration of clear, evidence-based recommendations. Our case contributes to the existing literature as an example of a congenital right-sided diaphragmatic hernia, illustrating the need of a CT-scan for accurate diagnosis, urgent surgical intervention when the symptoms are uncontrolled, and prosthetic reconstruction in case of a large defect.

Prevention or at least early anticipation of diaphragmatic hernias is more feasible in the pediatric population. Prenatal ultrasound allows the detection of the diaphragmatic hernias in 50% of the cases [9], knowing that isolated right-sided diaphragmatic defects are particularly challenging to diagnose [10]. In our patient, there was no suspicion of a diaphragmatic hernia which would have implied a long term follow-up [11]. However, a more rigorous surveillance could have been suggested given the anomalies observed during the laparotomy at the age of 1.

## Conclusion

Nontraumatic diaphragmatic hernia in an adult is rare but can be life-threatening. The primary challenges in these cases are maintaining clinical suspicion, especially when abdominal symptoms are absent, and determining the appropriate treatment strategy in the light of the rarity of the condition. Surgical intervention remains the only definitive treatment, typically performed via an abdominal approach with the use of a synthetic mesh to repair the defect.

## Author contributions

We both treated the patient, wrote, and reviewed the manuscript.

## Patient consent

The authors declare that written consent for publication was obtained from the patient.
